# Harnessing consumer smartphone and wearable sensors for clinical cancer research

**DOI:** 10.1038/s41746-020-00351-x

**Published:** 2020-10-27

**Authors:** Carissa A. Low

**Affiliations:** Department of Medicine, University of Pittsburgh, 3347 Forbes Avenue, Suite 200, Pittsburgh, PA 15213 USA

**Keywords:** Quality of life, Cancer, Risk factors, Palliative care

## Abstract

As smartphones and consumer wearable devices become more ubiquitous, there is a growing opportunity to capture rich mobile sensor data continuously, passively, and in real-world settings with minimal burden. In the context of cancer, changes in these passively sensed digital biomarkers may reflect meaningful variation in functional status, symptom burden, quality of life, and risk for adverse clinical outcomes. These data could enable real-time remote monitoring of patients between clinical encounters and more proactive, comprehensive, and personalized care. Over the past few years, small studies across a variety of cancer populations support the feasibility and potential clinical value of mobile sensors in oncology. Barriers to implementing mobile sensing in clinical oncology care include the challenges of managing and making sense of continuous sensor data, patient engagement issues, difficulty integrating sensor data into existing electronic health systems and clinical workflows, and ethical and privacy concerns. Multidisciplinary collaboration is needed to develop mobile sensing frameworks that overcome these barriers and that can be implemented at large-scale for remote monitoring of deteriorating health during or after cancer treatment or for promotion and tailoring of lifestyle or symptom management interventions. Leveraging digital technology has the potential to enrich scientific understanding of how cancer and its treatment affect patient lives, to use this understanding to offer more timely and personalized support to patients, and to improve clinical oncology outcomes.

## Introduction

Smartphones, owned by over 80% of US adults, provide new opportunities to understand and improve population health^1^. Many users keep their smartphones within arm’s reach at all times and spend hours per day interacting with the device^2^. Smartphones are equipped with a three-axis accelerometer, gyroscope, magnetometer, and other sensors capable of capturing moment-to-moment information about mobility, device use, social interactions, and environmental context as individuals go about their daily routines^3^. Smartwatches and other consumer wearable devices are also becoming more widely used^4^ and collect information about activity, sleep, and physiology. Thus, there is an unprecedented amount of health and behavioral data that can be captured continuously and passively using devices that many individuals already own.

For adults affected by cancer, mobile sensing can capture fluctuations in behavior that may reflect meaningful variation in functional status, symptom burden, quality of life, and risk for readmission and other adverse outcomes. Continuous assessment of these digital biomarkers^5^ could enable real-time monitoring of patients between clinical encounters, extending the coverage and reach of care. Further, sharing actionable insights from data analyses with providers, patients, and caregivers could lead to more proactive and personalized care. Although not without challenges, mobile sensing offers opportunities to increase patient engagement, remotely monitor patients at low cost, and evaluate the effects of treatments on patient functioning and daily activities.

Mobile sensor data can complement snapshots of health gathered during clinic visits as well as other forms of patient-generated health data^6^ such as patient-reported outcomes (PROs). Expanding use of PROs in clinical trials^7^ reflects growing awareness that PROs may improve capture of treatment toxicities (compared to clinician ratings, which tend to underestimate symptom severity and frequency^8^), helping patients make informed decisions and better prepare for what to expect during treatment. Measuring PROs systematically and between provider visits may also enable earlier symptom management, leading to better quality of life and potentially prolonged survival^9^. Similarly, measuring objective changes in physical activity, sleep, social interaction, and other passively sensed behaviors may improve and enrich our understanding of the effects of cancer and its treatments on daily life, support decision making and patient education, and enable earlier detection of deteriorating health and therefore earlier intervention. Unlike PROs, passive sensor data can be collected with minimal burden to patients, even during acute illness or over long periods of time, and reflect objective changes in behavior not subject to reporting biases.

This paper provides a narrative overview of the growing literature on digital biomarkers in clinical oncology, focusing specifically on studies linking consumer (as opposed to research-grade) wearable device or smartphone sensor data to clinical outcomes in cancer patients. To identify relevant papers, a search of the PubMed database was conducted in May 2020 using the following search terms: (smartphone OR mobile OR passive OR wearable) AND (sensors or sensing) AND (cancer OR oncology); (Fitbit OR Garmin OR Smartwatch) AND (cancer OR oncology). References of articles were also scanned for additional studies. After reviewing existing studies, challenges in integrating these data into routine oncology care as well as clinical applications of mobile sensing to improve comprehensive cancer care are discussed.

## Consumer wearable devices in oncology research

A vast and growing variety of consumer devices are available that are worn continuously on one’s body to quantify motion and other physiological factors (e.g., Fitbit, Apple Watch). These “wearable devices” include activity tracking wristbands or jewelry that are often paired with a companion smartphone app to sync and summarize a user’s data. Wearable devices are typically marketed as tools to motivate healthy lifestyles^4^. The wearable device market continues to evolve and expand, with some devices capable of measuring skin temperature and blood oxygen saturation or collecting electrocardiograms.

Consumer wearable devices are gaining traction in oncology research^10,11^. The most common uses of wearable devices in cancer research have been to assess physical activity (e.g., daily step counts) over time and to link activity metrics to outcomes of interest. Physical activity levels before, during, and after cancer treatment have been established as robust predictors of clinical outcomes as well as quality of life^12,13^. Compared with self-report assessments of physical activity, wearable devices offer more objective and fine-grained activity data^14^. Research generally supports the validity of commercial wearable devices for activity measurement^15^, although there is some evidence that step counts may be less accurate with slower gait speed or assistive device use^16^. Collection of activity data using consumer devices has been demonstrated to be feasible and acceptable among cancer patients in active treatment^17–20^. The wearable device literature builds on early studies with older-generation research-grade accelerometers^21^, which have been shown to correlate with consumer activity tracker data in cancer patients^22^. Unlike research-grade actigraphy devices, which are costly, require specialized software to access and analyze the data, and are commonly worn for a week or less, consumer wearable devices are affordable and designed to be comfortable and convenient to wear continuously with easily recharged batteries that enable long-term data collection. Another major advantage of commercial devices is that they frequently sync data to cloud-based servers, enabling remote real-time monitoring of activity data by researchers or clinicians. Thus, wearable devices provide a way to collect physical activity and other behavioral and physiological data continuously, unobtrusively, and at large scale in free-living conditions^23^.

Prospective observational studies support the hypothesis that physical activity quantified by consumer wearable devices is correlated with symptom burden and quality of life. A study of 32 patients undergoing hematopoietic cell transplant found that lower Fitbit step counts were associated with higher levels of self-reported symptoms including pain, fatigue, and diarrhea as well as worse physical health and role functioning (but not mental health or quality of life)^24^. Furthermore, within-person decreases in daily steps were associated with increasing symptoms and worsening physical health over time. In a study of 39 patients with advanced lung cancer, lower Fitbit step counts were correlated with lower quality of life and greater depression^25^. A study of 24 patients in systemic therapy found that Fitbit step count variables were correlated with patient-reported fatigue, quality of life, depression, and performance status^26^. These studies suggest that low or decreasing activity may indicate worsening symptoms and may be a sensitive measure of performance status.

Studies have also shown that wearable device data relate to clinical outcomes. During outpatient chemoradiation therapy, lower levels of activity as measured with Garmin devices were associated with greater hospitalization risk, lower likelihood of completing treatment without delays, and shorter survival^27,28^. In a sample of 71 patients undergoing surgery for advanced abdominal cancer, lower Fitbit step counts during inpatient recovery were associated with greater risk of unplanned 30- and 60-day hospital readmission^29^. A study of 20 abdominal cancer patients reported that daily step count on postoperative day 7 inversely correlated with postoperative complication index^30^. Finally, a study of 37 advanced cancer patients found that higher daily Fitbit step counts were associated with better provider-assessed performance status as well as reduced odds of hospitalization and death^31^. Thus, remote real-time monitoring of activity data may identify patients at risk for adverse outcomes who could benefit from additional support or intervention.

Because wearable devices offer data and feedback directly to patients, these tools are also used to promote physical activity among cancer patients and survivors^32^. Two recent reviews of studies using wearable devices to promote activity among cancer survivors found that interventions using wearable activity monitors had a positive impact on physical activity as well as symptoms and quality of life^33,34^. Moreover, real-time wearable device data may allow for tailored or just-in-time physical activity interventions^35^ that may be further enhanced by the support of a coach who can review a patient’s real-time data and provide individualized support for behavior change^36^.

Although many wearable devices also measure sleep and heart rate in addition to activity, to date there is limited published evidence linking these metrics to patient-reported symptoms, quality of life, or outcomes.

## Smartphone sensors in oncology research

Unlike wearable devices, which are owned by only 21% of adults and an even smaller percentage of older or rural populations, the vast majority of adults in the United States already own and use a smartphone^1^. This ubiquity makes research or clinical applications using smartphones highly scalable. Like wearable devices, smartphones are equipped with onboard accelerometer sensors as well as other sensors capable of passively measuring ambient light and noise, battery level, whether the screen is on or off, nearby Bluetooth devices, and other parameters related to individual behavior patterns and environmental context. For example, location data may shed light on how much time is spent at home, at work, in the hospital, or in social settings. Speed of movement may be related to depression or fatigue, whereas frequency of smartphone screen unlocks may be related to concentration or anxiety^37^. Leveraging data from these sensors for what has been called digital phenotyping is prevalent in the field of mental health^38–40^ but less established in oncology.

In the past few years, several studies suggest that smartphones may have clinical utility for oncology care. Some of these have focused exclusively on the ability of smartphone accelerometers to estimate step count, reporting that smartphone measurements are valid and reliable^41^. A pilot intervention where chemotherapy patients were given a smartphone with a pedometer application and contacted if their daily step count decreased >15% from baseline was shown to be feasible and to help identify chemotherapy toxicity^42^. In a prospective study of 62 patients undergoing cancer surgery, mean exertional activity based on smartphone accelerometer data was useful in differentiating patients who experienced a postoperative complication, emergency department visit, readmission, reoperation, or mortality from those with better recovery trajectories^43^.

Four pilot studies have harnessed smartphone sensors beyond accelerometry to monitor cancer patients, sometimes in combination with wearable devices. In a sample of 14 patients undergoing chemotherapy for gastrointestinal cancer, machine learning models using smartphone sensor data like screen time and location in combination with Fitbit data were able to detect symptom burden with 87% accuracy^44^. The subset of features yielding the best performing model included more time in frequent locations, fewer minutes in light physical activity, and more time interacting with the device. In a sample of seven breast cancer patients in active treatment, more time at home or at locations of friends and family members was associated with better mood and less anxiety and depression while more time spent at hospitals and clinics was associated with worse mood^45^. In a sample of 10 gynecologic cancer patients, Fitbit and smartphone sensor data were actively monitored for anomalies; this led the research team to identify a patient with severe nausea and vomiting whose symptoms were then able to be managed over the phone as well as a patient who took fewer steps and spent less time away from home prior to an emergency department visit^46^. Finally, a recent study collected both smartphone and wearable sensor data as well as patient-reported pain and distress ratings and quality of life measures in 25 palliative cancer patients^47^. Mobile sensor data were significantly correlated with pain and distress ratings. Moreover, although patient-reported pain, distress, and quality of life were not associated with emergency visits, increased resting heart rate, decreased heart rate variability, and a trend for increased step speed was associated with emergency visits. These last two studies provide preliminary evidence that anomalies in passive sensor data, particularly deviations in heart rate and activity data, may serve as early warning signals for clinically significant deterioration, presaging changes in patient self-report and offering a wider window for preventive intervention.

## Other sensors in oncology research

This review focuses on personal consumer devices and passively captured data from typical patterns of use, but there are many opportunities for additional passive sensors or applications using existing sensors to inform oncology care. For example, wearable or smartphone sensors can be used in performance-based tasks such as the six-minute walk test or 30-second maximal sit-to-stand test^48,49^. Additional sensors such as Bluetooth scales or blood pressure monitors or beacons to estimate in-home location may provide additional data about health status, home environment, and activities of daily living^50,51^. Passive monitoring of diet or eating behaviors has proven challenging, although using smartphone cameras with machine learning algorithms to label foods and estimate portions may be less burdensome than manually entering foods^6^. Research is underway investigating wearable sensors to detect biomarkers in sweat^52^ and ingestible sensors to monitor medication compliance^53^. Finally, sensing opportunities related to cancer prevention include leveraging wearable sensor data to detect smoking gestures^54^ or using wearable ultraviolet radiation sensors to monitor sun exposure^55^.

## Summary of previous research

Over the past four years, a growing body of evidence supports the feasibility and potential clinical value of mobile sensors in clinical oncology research. We identified 14 prospective studies since 2016 that have examined a variety of oncology populations before, during, and after cancer treatment and have included a range of consumer wearable devices and smartphone sensing frameworks (see Table 1). Most studies have included very small samples (median *n* = 34.5, range 7–71), and only a handful have used real-time sensor data to inform clinical care^42,46^. Larger-scale longitudinal studies that implement mobile sensing in clinical settings are needed.

**Table 1:** Prospective studies examining mobile sensor data and clinical outcomes in oncology patients.

Author and year^ref^	Number of patients	Mean/median age	Type of patients	Device(s) used	Timing	Outcome
Bennett et al.^24^	32	55	Hematopoietic stem cell transplant	Fitbit Flex	During transplant hospitalization and 4 weeks post-discharge	PRO-CTCAE for toxicities and symptoms and PROMIS physical health, mental health, and quality of life
Bade et al.^25^	39	66	Advanced lung cancer	Fitbit Zip	For 7 days at varying points in treatment	EORTC QLQ-30 quality of life, PHQ-9 depressive symptoms, MMRC dyspnea scale
Gupta et al.^26^	24	54	Mixed cancer	Fitbit Flex	12 weeks during systemic therapy	ECOG Performance Status, FACT-G quality of life, QIDS-SR16 depressive symptoms, BFI fatigue
Ohri et al.^27^	50	66	Locally advanced non-small cell lung cancer	Garmin Vivofit	Before start of chemoradiation therapy to completion of the first week (median 17 days)	Hospitalization during radiation, completion of radiation without delay, progression-free and overall survival
Ohri et al.^28^	38	64	Head and neck, lung, and gastrointestinal cancer	Garmin	During concurrent chemoradiotherapy	Unplanned hospitalization during treatment
Low et al.^29^	71	57	Metastatic peritoneal cancer	Fitbit Flex or Charge	During inpatient recovery from cytoreductive surgery with hyperthermic intraperitoneal chemotherapy	30- and 60-day readmission
Sun et al.^30^	20	56	Hepatobiliary and gastrointestinal cancer	Garmin Vivovit 2	3–7 days before surgery, during inpatient recovery, and up to two weeks following discharge	30-day comprehensive complication Index based on Clavien-Dindo classification
Gresham et al.^31^	37	62	Mixed advanced cancer	Fitbit Charge HR	14 days during or awaiting chemotherapy or radiation therapy	Provider-assessed ECOG & Karnofsky performance status, adverse events, hospitalizations, 6-month survival
Soto-Perez-de-Delis et al.^42^	40	73	Mixed solid tumors	Android smartphone with Google Fit pedometer app	Before (median 11 days) and during (median 21 days) chemotherapy	Grade 2 or 3 toxicities (commonly fatigue, nausea/vomiting, diarrhea, infections)
Panda et al.^43^	62	66	Mixed cancer	iOS or Android smartphone with Beiwe sensing app	Before (median 5 days) and after (median 147 days) cancer surgery	Clinically significant postoperative events
Low et al.^44^	14	60	Gastrointestinal cancer	Android smartphone with AWARE sensing app and Fitbit charge HR	Four weeks during chemotherapy	Daily symptom burden
Cai et al.^45^	7	60	Breast cancer	iOS or Android smartphone with Sensus sensing app	Seven weeks during active treatment	Daily and weekly mood, anxiety, and depression ratings
Wright et al.^46^	10	60	Gynecologic cancer	iOS or Android smartphone with Beiwe sensing app, Fitbit Zip and Charge 2	30 days during chemotherapy	Daily PRO-CTCAE toxicities and PROMIS quality of life
Pavic et al.^47^	30	64	Metastatic cancer	Everion bracelet by Biovotion plus Samsung Galaxy S5	12 weeks starting at discharge from the hospital	Emergency visits, readmissions, and deaths

## Challenges and practical considerations

Several issues must be considered when integrating mobile sensing into cancer research and care. Addressing these challenges will enable broader use of these methods and allow cancer researchers and clinicians to capitalize on mobile technology advances and maximize potential benefit to patients and families.

The first set of challenges involves the collection, cleaning, and processing of mobile sensor data to make it useful to patients or providers. One hurdle is selecting the right device to use, as existing evidence provides only limited guidance regarding which digital biomarkers may be most valuable for specific clinical oncology populations or contexts. Selecting which device to use may be a function of feasibility and cost of data collection, where and for how long data will be collected, and whether real-time data will be monitored. For example, some wearable devices are uncomfortable to wear overnight or require daily charging and therefore may not be the ideal choice for a long-term sleep monitoring study. Harnessing data from whichever device patients already own is appealing, but it remains unclear whether metrics of activity, sleep, and other behaviors are comparable across different manufacturer’s devices, and it may be challenging to standardize data collection, compilation, and processing across different devices. As the devices discussed in this review were developed as consumer devices rather than research-grade measures, additional work is needed to validate that sensor data reliably measure behaviors of interest compared with gold-standard measures, evaluate their robustness in real-world settings, and confirm their correlation with clinical outcomes in older cancer populations.

Translating raw data into digital biomarkers requires multidisciplinary collaboration, as the volume of continuously collected sensor data can be enormous, and both clinical and technical expertise are needed to extract meaning from continuous sensor data. Some data reduction such as calculation of daily step counts or average resting heart rate may be handled by device software through opaque proprietary algorithms^56^. To fully realize the potential of continuous sensor data, researchers may want to consider whether raw or more granular (e.g., minute-level step count, beat-to-beat heart rate, continuous location) data are available so that they can calculate additional features like gait speed, percent of time spent at home, circadian rhythmicity of activity, or variability in heart rate^37^. Interviews with patients, providers, and other stakeholders may be useful in identifying additional useful and relevant biomarkers^57^. Other decisions include how to handle missing data, time windows over which to aggregate data, and which data analytic approach to use. Missing sensor data are common owing to both participant noncompliance and technical issues, and guidelines for best practices in cleaning data (e.g., deleting duplicates or handling outliers) or considering issues like how to determine when a participant was wearing a device and the minimum amount of daily wear time necessary to estimate activity level have not yet been established^10^. From a relatively modest number of sensors, hundreds of features can be extracted from single sensors or combinations of sensors (e.g., high heart rate during sedentary behavior). With such a large number of input variables that may contain predictive value, finding robust patterns linking these features to clinical outcomes may require machine learning approaches that can be complex, computationally intensive, and difficult to interpret. Visualizing data in an accessible way for patients, caregivers, researchers, or clinical providers requires user-centered design to better understand each stakeholder’s needs and preferences and to determine how to transform data analyses into actionable recommendations^58^.

A second set of issues involve health system and patient barriers to mobile-sensing implementation. For mobile sensor data and associated prediction models to be clinically useful, seamless integration with electronic health records (EHR) and existing workflows would be ideal. Pulling data collected by patients’ personal wearable devices or smartphones into EHR would allow these data to be viewed and discussed during clinical encounters and would also enable automatic provider alerts to be triggered if prespecified thresholds are exceeded or significant anomalies are detected^59^. Making data available to clinicians and integrated into their EHR workflow will require training or guidance about how to use these data in clinical conversations and decision making^60^. In one feasibility trial that integrated Fitbit step count data into EHR, investigators reported that the majority of clinicians looked at survivors’ activity data and reported that Fitbit data provided insight into their patients’ lifestyles^61^.

Even with passive sensing, patient engagement is critical in ensuring quality and clinical utility of patient-generated health data. If patients are required to wear, sync, or charge devices consistently, frequent reminders or technical support may be necessary. If data from personal smartphones is being shared with providers or researchers, patients must fully understand and consent to this data sharing. If patients have difficulty adhering to recommended use or if compliance declines over time, interviews to understand barriers to using the technology and whether patients believe sensor data are valid and useful may be enlightening^10^.

Third, ethical challenges must be confronted in using mobile sensor data^62,63^. These include ensuring that patients understand the risks and benefits of collecting and sharing these data and that risks related to privacy and data security are minimized. It is also important to ensure that advances in mobile-sensing tools are accessible to diverse populations. Smartphone use is widespread across racial and economic groups, but wearable devices are more common among individuals of higher socioeconomic status^4^. Digital literacy may be particularly relevant for older cancer patients, who may own smartphones or wearable devices but not understand how to fully utilize their capabilities. It will be important to consider issues like access to technology and consistent Wi-Fi for data syncing as well as digital literacy to prevent these innovations from widening existing health disparities.

## Opportunities and clinical implications

**Fig. 1:**
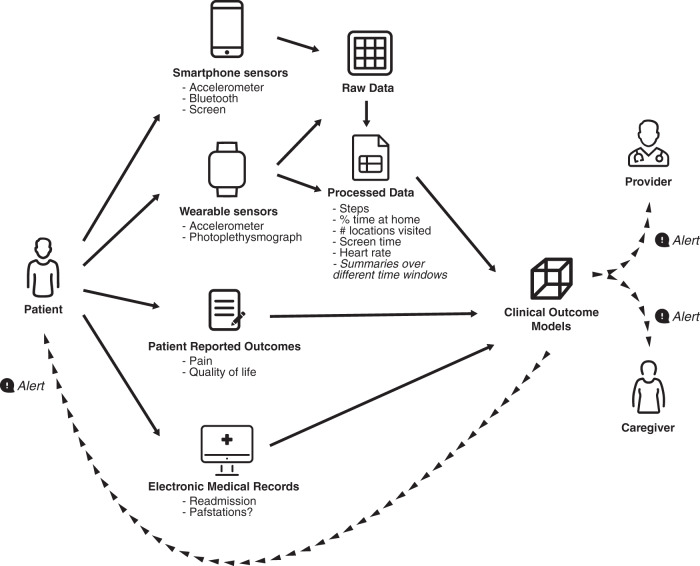
Highlights a mobile sensing framework for monitoring oncology patients. Patients provide passive sensor data via smartphone and wearable sensors that, when combined with patient-reported outcomes or electronic health record data, can be used to generate clinical risk prediction models. These models can be used to trigger alerts for oncology providers or alerts for personalized interventions for patients and caregivers.

There are a number of potential clinical applications for smartphone and wearable sensors in clinical oncology (Fig. 1). Behavioral sensor data could be useful in evaluating patients prior to treatment to obtain a more objective measure of performance status or frailty and to predict likelihood of tolerating a new or aggressive therapy^58,64^. Digital biomarkers could also be included as secondary measures of performance status in clinical trials, providing many more data points from which to gain insight about the effect of treatments on patient lives^56,57^. If mobile sensing data are monitored in real-time or used in combination with algorithms that trigger notifications to clinicians when preset thresholds are exceeded or trajectories suggest significant anomalies or changes over time, they can be used for remote monitoring of toxicities and symptoms^57,65^. These symptom monitoring systems could also trigger symptom self-management interventions for patients and caregivers or support patients in making timely decisions about when to seek care^42,46^. In the post-treatment survivorship phase, when clinical encounters become less frequent but treatment sequelae like fatigue, insomnia, and cognitive difficulties remain common, passive monitoring could be useful in evaluating quality of life, personalizing survivorship care, and gaining insight into the long-term effects of cancer and its treatments^64^.

Mobile sensor data can also be used to inform, trigger, or personalize supportive interventions. For example, wearable devices can be used to monitor and motivate physical activity as part of prehabilitation or rehabilitation efforts. Interventions such as those to support pain management, address cognitive impairment, or reduce stress can be delivered via smartphone apps, and collecting concurrent sensor data can enable researchers to evaluate app use patterns and behavioral changes in response to intervention content^45,60^. Furthermore, sensor data can be used to tailor intervention content, dose, or timing, setting attainable individualized goals and delivering prompts at the right time, when participants are receptive to intervention messages or in need of support^63^. Finally, sensor data themselves can be leveraged to develop n-of-1 interventions, helping individual patients identify patterns and recognize how their activity, sleep, or other behaviors affect their health.

## Conclusion

Smartphones and wearable devices are becoming ubiquitous and offer powerful opportunities for passive remote sensing of patient behavior and health. Existing data based on small observational studies suggest that collecting mobile sensor data from cancer patients is feasible and that these digital biomarkers are related to symptoms, quality of life, and physical function as well as risk of adverse events. Monitoring mobile sensor data in real-time between clinical encounters may lead to earlier detection of significant changes in activity, sleep, social interactions, or other behaviors, allowing more proactive and timely management of toxicities and risks during and after cancer treatment. Mobile sensing can also be used to promote and personalize behavior change recommendations, such as lifestyle or symptom management interventions. Implementing mobile sensing in oncology clinics will require multidisciplinary teams to support the management and interpretation of large amounts of continuous data collected by mobile devices, consideration of ethical and privacy issues, and engagement with patients, providers, EHR systems, and other stakeholders in order to minimize barriers to uptake. Leveraging these ubiquitous technologies has the potential to improve scientific understanding of how cancer and its treatment affect patient lives and to use this understanding to better support and empower patients, personalize and extend care, and improve clinical outcomes.

## Data Availability

No data sets were generated or analyzed during the current study.
